# Temporal Effects on Internal Fluorescence Emissions Associated with Aflatoxin Contamination from Corn Kernel Cross-Sections Inoculated with Toxigenic and Atoxigenic *Aspergillus flavus*

**DOI:** 10.3389/fmicb.2017.01718

**Published:** 2017-09-15

**Authors:** Zuzana Hruska, Haibo Yao, Russell Kincaid, Robert L. Brown, Deepak Bhatnagar, Thomas E. Cleveland

**Affiliations:** ^1^Geosystems Research Institute, Stennis Space Center, Mississippi State University, Starkville MS, United States; ^2^Southern Regional Research Center, United States Department of Agriculture-Agricultural Research Service, New Orleans LA, United States

**Keywords:** aflatoxin, *Aspergillus flavus*, internal fluorescence, hyperspectral imaging, maize

## Abstract

Non-invasive, easy to use and cost-effective technology offers a valuable alternative for rapid detection of carcinogenic fungal metabolites, namely aflatoxins, in commodities. One relatively recent development in this area is the use of spectral technology. Fluorescence hyperspectral imaging, in particular, offers a potential rapid and non-invasive method for detecting the presence of aflatoxins in maize infected with the toxigenic fungus *Aspergillus flavus*. Earlier studies have shown that whole maize kernels contaminated with aflatoxins exhibit different spectral signatures from uncontaminated kernels based on the external fluorescence emission of the whole kernels. Here, the effect of time on the internal fluorescence spectral emissions from cross-sections of kernels infected with toxigenic and atoxigenic *A. flavus*, were examined in order to elucidate the interaction between the fluorescence signals emitted by some aflatoxin contaminated maize kernels and the fungal invasion resulting in the production of aflatoxins. First, the difference in internal fluorescence emissions between cross-sections of kernels incubated in toxigenic and atoxigenic inoculum was assessed. Kernels were inoculated with each strain for 5, 7, and 9 days before cross-sectioning and imaging. There were 270 kernels (540 halves) imaged, including controls. Second, in a different set of kernels (15 kernels/group; 135 total), the germ of each kernel was separated from the endosperm to determine the major areas of aflatoxin accumulation and progression over nine growth days. Kernels were inoculated with toxigenic and atoxigenic fungal strains for 5, 7, and 9 days before the endosperm and germ were separated, followed by fluorescence hyperspectral imaging and chemical aflatoxin determination. A marked difference in fluorescence intensity was shown between the toxigenic and atoxigenic strains on day nine post-inoculation, which may be a useful indicator of the location of aflatoxin contamination. This finding suggests that both, the fluorescence peak shift and intensity as well as timing, may be essential in distinguishing toxigenic and atoxigenic fungi based on spectral features. Results also reveal a possible preferential difference in the internal colonization of maize kernels between the toxigenic and atoxigenic strains of *A. flavus* suggesting a potential window for differentiating the strains based on fluorescence spectra at specific time points.

## Introduction

Invasion of commodities by mycotoxin producing fungi is a common problem faced by farmers around the world predominantly in tropical and subtropical climates. Aflatoxins are the most carcinogenic of all the mycotoxins, adversely affecting human and animal populations through ingestion or inhalation of contaminated food and feed (Richard and Payne, 2003). Crops most commonly affected by aflatoxin contamination are oilseeds including maize, cottonseed, and tree and ground nuts. While most industrialized countries have controls (legislation) in place to limit aflatoxin entering the food stream during years with environmental conditions (high heat and humidity) prone to accelerated aflatoxin production, many developing countries are lagging behind (Van Egmond, 2002; Van Egmond and Jonker, 2005; Van Egmond et al., 2007). Additionally, global weather shifts expand areas susceptible to aflatoxin contamination (e.g., Africa, Asia, and North Australia), which now include also more temperate parts of the world (parts of Europe and United States). Therefore, a timely solution is needed to curb this problem and prevent the situation from reaching critical proportions (Wu et al., 2011; Battilani et al., 2016).

Although most aflatoxin contamination of crops originates pre-harvest, much of the problem is attributed to less than optimal post-harvest handling and storage. For this reason, it is important to detect and remove the contaminated ears/seed early, so that spread during storage can be prevented. Detection of the contaminant is still largely dependent on costly laboratory instrumentation techniques, which are quite sophisticated and able to determine several mycotoxins simultaneously with high precision (Wacoo et al., 2014). Unfortunately, while advanced, these systems/methods lack the practicality required for outdoor applications (e.g., field and market). The recent research focus on rapid and non-invasive technology offers alternative possibilities for detecting aflatoxins and other mycotoxins in staple commodities including maize and groundnuts.

One of the rapid and non-invasive systems, fluorescence hyperspectral imaging technology, was primarily developed for food and feed inspection/safety applications (Kim et al., 2001; Zavattini et al., 2004). A fluorescence hyperspectral imaging system is typically based on an imaging spectrometer or hyperspectral imager coupled with a UV lamp or a laser, which enables the acquisition of fluorescence image data with both high spectral and spatial resolutions. Fluorescence results from exciting a target (e.g., maize kernels) with a short wavelength illumination (e.g., UV) and collecting the absorbed emissions from the target material at a longer wavelength. Fluorescence hyperspectral imaging has been shown to be effective for quality and safety evaluation of various agricultural products (reviewed by Feng and Sun, 2012; Zhang et al., 2012; Huang et al., 2014).

Detecting aflatoxin in contaminated maize based on fluorescence was extensively explored in the 1970s and 1980s (Fennell et al., 1973; Lillehoj et al., 1976; Lee et al., 1980). An established relationship between bright greenish yellow fluorescence (BGYF) and cotton seed/fiber was noted even earlier (Marsh et al., 1969). These early observations were the basis of the presumptive “black light” (365 nm UV) test for detecting potential fungal/aflatoxin contamination in cotton seed, and later, in maize (Bothast and Hesseltine, 1975; Shotwell and Hesseltine, 1981), pecans (Tyson and Clark, 1974), and pistachio nuts (Hadavi, 2005). Early on, it was realized that the association between the BGYF and aflatoxin found in cotton did not translate to the same degree in maize (Lee et al., 1980). The correlation in maize was minimal and the association was sporadic. That is, some fluorescing kernels exhibited the expected high aflatoxin content, but others did not (Lillehoj et al., 1976). Additionally, some non-fluorescing kernels were highly contaminated (Fennell et al., 1973). This unpredictability made the test unreliable as a definitive detection tool for maize, but remained as a presumptive, screening test indicating a potential aflatoxin contamination and the need for further analysis. Several additional studies demonstrated the presence of BGYF on and in kernels infected with aflatoxins and explained the fluorescence to be due to the presence of a BGYF compound formed by the oxidizing effect of peroxidases in live plant tissue acting on kojic acid, another secondary metabolite of *Aspergillus flavus* formed with aflatoxin (Wicklow, 1999). The problem relating to inaccurate detection of aflatoxin using the indirect BGYF method arises when the BGYF appears in kernels that are infected with *A. flavus* strains that produce kojic acid, but not aflatoxins, resulting in a false positive test possibly due to the presence of non-aflatoxigenic fungi (Wicklow et al., 1998; Wicklow, 1999), or when intact non-BGYF kernels contain aflatoxin and are, therefore, false negatives.

The black light test elicits the fluorescence signal, whereas hyperspectral imaging measures the fluorescence output within a discreet spectral range and allows the comparison and quantification of treatment effects. The use of fluorescence hyperspectral imaging in the attempt to detect aflatoxin contamination in maize, revealed a spectral peak shift between contaminated and uncontaminated kernels (Yao et al., 2010, 2013). The fluorescence peak of the contaminated kernels consistently shifted to higher wavelengths from the uncontaminated controls. The shift is usually associated with BGY or BY (bright yellow) fluorescing kernels, but not always. In some instances, clean looking, non-fluorescing kernels do exhibit a shift towards a longer wavelength and test aflatoxin-positive when chemically analyzed (Hruska et al., 2016, unpublished data). It appears that a significant portion of aflatoxin contamination of maize is contained in intact kernels that exhibit internal BGY fluorescence only apparent once the kernel is crushed (Shotwell et al., 1974). Also, naturally occurring populations of fungi that colonize maize may at any given time consist of a variety of *A. flavus* strains, toxigenic, and atoxigenic, both potentially exhibiting BGYF (Wicklow, 1999).

The interaction between the fluorescence signals emitted by some aflatoxin contaminated maize kernels and the fungal invasion resulting in the production of aflatoxins has not been fully elucidated. In order to gain a deeper insight into the problem, the objective of the present study was to examine the internal fluorescence spectral emissions from cross-sections of kernels infected with toxigenic and atoxigenic strains of *A. flavus* with the aid of fluorescence hyperspectral imaging technology over a 9 day incubation period. Additionally, the germ of each kernel was separated from the endosperm in order to determine the difference in fungal invasion in the major areas of maize kernels, and the consequent accumulation and progression of aflatoxins in these regions, over time.

## Materials and Methods

### Fungal Strains and Preparation of Inocula

Two fungal isolates, an aflatoxin producing AF13-SRRC 1532, and a non-aflatoxigenic AF36, were obtained from the Food and Feed Safety laboratory (FFS), SRRC, Agricultural Research Service (ARS), USDA, and cultured on potato dextrose agar (PDA) medium in plastic Petri dishes at 30 ± 1°C in the dark. Conidia were harvested on fifth day of growth and suspended in buffer at a dilution of 5 × 10^6^ conidia/mL, determined with a haemocytometer. The inocula were stored in separate containers at 4 ± 1°C.

### Sample Preparation – *In Situ* Inoculation

Maize kernels (N78B-GT, Syngenta NK Brand Seeds, Laurinburg, NC, United States), utilized in the current experiments were harvested in 2010 from the ARS Field Station in Stoneville, MS, United States. Whole, undamaged kernels of roughly uniform size were randomly assigned into three treatment groups and processed according to a modified kernel screening assay developed for laboratory inoculation experiments (KSA; Brown et al., 1995). All kernels were surface sterilized in 70% ethanol and rinsed in three changes of dH_2_O. Kernels in each treatment group were inoculated by immersion and stirred for 1 min. The treatments included: (1) kernels inoculated with AF13, (2) kernels inoculated with AF36, and (3) kernels inoculated only with dH_2_O as control for non-specific fluorescence. Each group of kernels was incubated in a humidity chamber using a plastic tray with individual compartments totaling approximately 200 inoculated kernels per treatment, per imaging time point (growth days 5, 7, and 9). Distilled water was added every other day to each incubation chamber in order to maintain constant moisture. Kernels were incubated at 30 ± 1°C and examined at several time points (5, 7, and 9 days) post-inoculation. A subset of the original treatment pool was used in the imaging experiments.

### Experimental Procedures

#### Experiment 1

**FIGURE 1:**
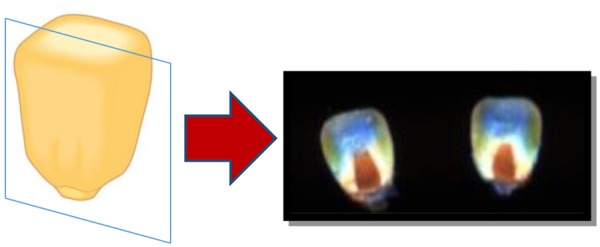
Cross-sections were achieved by slicing each kernel vertically on a frontal plane. All cross-sections were imaged cut side up.

**FIGURE 2:**
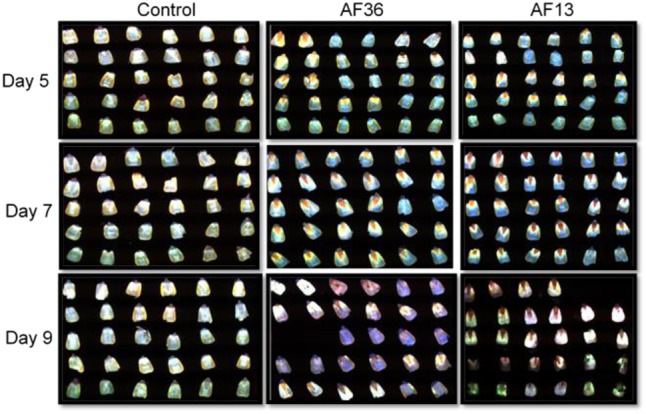
Fluorescence images of maize kernel cross-sections inoculated with toxigenic and atoxigenic strains of *Aspergillus flavus* over a 9 day growth period, including controls. Due to extensive deterioration, two kernels from the two treated groups (AF13 and AF36) on day 9, were excluded from analysis.

At each specified time interval (Days 5, 7, and 9), 30 maize kernels per treatment group (AF13, AF36, and Control) were removed from the incubator, wiped free of visible external mold growth, sliced vertically on a frontal plane (**Figure 1**), and placed in a dark imaging dish, 15 kernels (30 halves) per image. Ninety kernels (180 halves) in each group were imaged cut side up over 3 days with a fluorescence hyperspectral sensor, 270 kernels (540 halves) overall. Few treated kernels from day 9 were badly deteriorated and thus were excluded from analysis. Representative images from each day are presented in **Figure 2**.

#### Experiment 2

**FIGURE 3:**
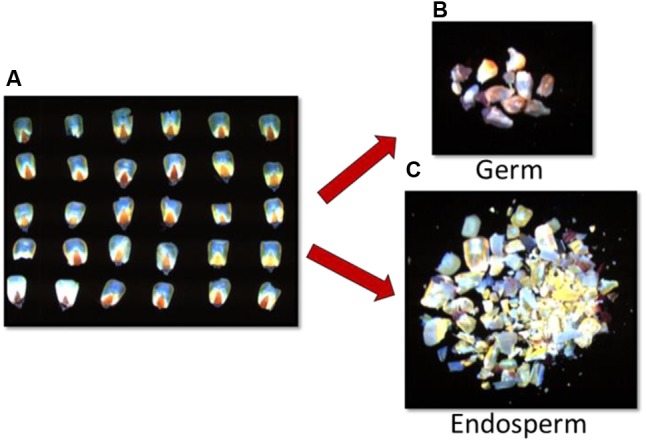
Fluorescence images of **(A)** corn kernel cross-sections inoculated with toxigenic and atoxigenic strains of *A. flavus* (AF13 and AF36), separated and grouped into **(B)** germ and **(C)** endosperm.

A second set of inoculated and control kernels from three time intervals (Days 5, 7, and 9), were used to determine the major area of fungal invasion, and aflatoxin accumulation/progression over nine growth days by separating the germ from the endosperm of each kernel prior to imaging. Fifteen kernels from each treatment group per day (135 total), were cross-sectioned with a razor blade and the embryos (germ) were scooped out and pooled for a single image. The remaining endosperms from all kernels were also pooled and imaged (**Figure 3**). After imaging, each pooled sample was processed for aflatoxin determination.

### Fluorescence Hyperspectral Imaging

Images were acquired with a push-broom, line scanning fluorescence hyperspectral imaging system (VNIR 100E, Stennis Space Center, Hancock County, MS, United States) under UV excitation, using a long wave (UV-A) ultraviolet (UV) lamp assembly (Model XX-15A) from Fisher Scientific (Thermo Fisher Scientific Inc., Waltham, MA, United States) with wavelength centered at 365 nm and a power output of 196 mw (Yao et al., 2010). The target for single kernel imaging of the cross-sections was a rectangular ceramic tray with 30 shallow indentations, each large enough to accommodate a single corn kernel. The grouped kernel constituents were imaged on a round ceramic tray approximately 10 cm in diameter. Both trays were painted with a flat, black paint to reduce the reflected light reaching the sensor and also to enhance the contrast between the background and the samples. A dark current image, acquired with the lens cap on, was also taken for calibration purposes.

### Image Processing

Image preprocessing of raw hyperspectral images included sensor background noise removal through dark current subtraction, image band wavelength assignment, and random noise removal via Savitzky–Golay filtering. Each preprocessed image had a spectral range from 400 to 900 nm with a total of 175 bands. The spatial size of each image was 800 × 425 pixels. The final number of images was 560, which totaled 124 Gbytes. Each image consisted of two components, corn kernels and background. A spectral threshold process was used to build a mask image with individual corn kernels or grouped germ and endosperm in the foreground, and the plate in the background. The threshold process used one image band of the fluorescence hyperspectral image. The mask image was applied to the corresponding hyperspectral image to create a region of interest (ROI) of each kernel on the tray. Spectral fluorescence of each target (e.g., single kernel, grouped germs, etc.) was averaged and extracted from the whole tray ROI. Further image analysis was implemented within only these ROIs.

### Aflatoxin Determination (Experiment 2)

Imaged kernel components (germ and endosperm) were dried in a 60 ± 1°C oven for 2 days and chemically analyzed for aflatoxin. The AflaTest (VICAM, Milford, MA, United States) method, approved for quantitative analysis of aflatoxin in maize by the United States Department of Agriculture Federal Grain Inspection Service, was used to determine aflatoxin concentrations in the maize samples. Details of the method were described previously (Hruska et al., 2013). Briefly, samples were weighed, crushed and extracted with ACS reagent grade methanol (Fisher Scientific) in water 80/20 (v/v). Extracts were diluted, filtered, and passed through AflaTest columns. The columns were washed with distilled water and eluted with 100% methanol. Eluted samples were mixed with developer (1:1) and aflatoxin (parts per billion, ppb) was measured in the EX-4 series Fluorometer (VICAM).

### Statistical Analysis

#### Spectral Data

Spectral peak shifts of maize kernel cross-sections (Experiment 1) and subset germ and endosperm areas (Experiment 2) were analyzed for group differences with one-way analysis of variance (ANOVA) using Matlab 2014, and graphed with Microsoft Office Excel 2010 software.

#### Chemical Data (Experiment 2)

Because all 15 samples in each group were pooled and analyzed as a single sample, the differences in aflatoxin concentration between germ and endosperm samples are illustrated graphically. Error bars represent % differences.

## Results

### Image Data – Internal Fluorescence of Corn Cross-Sections

**FIGURE 4:**
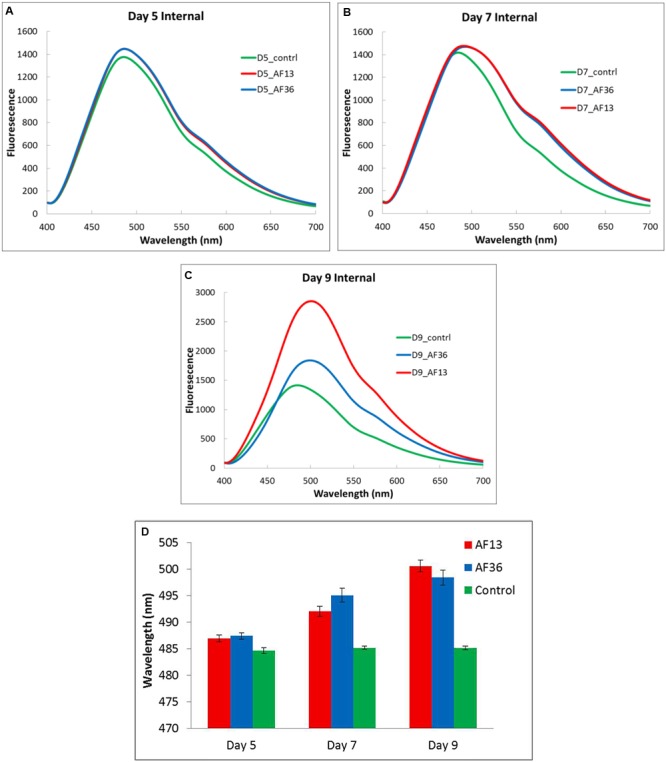
Internal fluorescence spectral means **(A–C)** and analysis of variance between fluorescence peak means ± SE **(D)**, from cross-sections of maize kernels inoculated with toxigenic and atoxigenic strains of *A. flavus* (AF13 and AF36) at three time points over a 9 day period (**A**-Day 5, **B**-Day 7, and **C**-Day 9), including controls.

The spectral means of the cross-section internal fluorescence from maize kernels incubated in toxigenic and atoxigenic *A. flavus* inoculi at three time points over a 9 day period (Days 5, 7, and 9) are presented in **Figures 4A–C**. Imaging data were statistically analyzed to determine the existence of group differences in spectral peak shifts between the two fungal inoculi and the controls (**Figure 4D**). Significant treatment effect, *p* < 0.01, was revealed on day 5 of incubation between spectral peaks of both treatment groups and the controls. This effect persisted and increased (*p* < 0.001) through day 9. There was no significant difference observed between peak shifts of the two treatment groups (AF13 and AF36) over the treatment period. The results indicate and confirm previous results found with intact kernels (data not shown), that it is not possible to differentiate aflatoxin producing *A. flavus* from the non-producing strain in maize cross-sections, on the basis of a fluorescence peak shift. There was, however, a marked difference in intensity indicating a significantly brighter fluorescence emitting from the kernels treated with the toxin producing AF13 compared to those treated with the atoxigenic AF36 fungal strain, on day 9 post-inoculation (**Figure 4C**). It may be worth noting that the controlled laboratory inoculation time frame with the corresponding spectral changes may not reflect infection, natural or artificial, in the field.

### Image Data – Fluorescence of Germ and Endosperm

**FIGURE 5:**
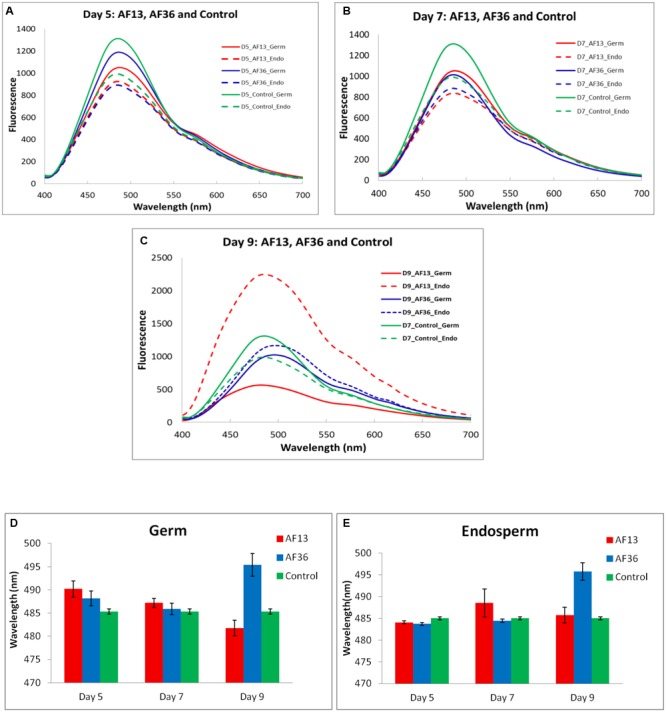
Fluorescence spectral means **(A–C)** and analysis of variance between fluorescence peak means ± SE of maize kernel components (**D**-germ and **E**-endosperm), inoculated with toxigenic and atoxigenic strains of *A. flavus* (AF13 and AF36) at three time points over a 9 day period (**A**-Day 5, **B**-Day 7, and **C**-Day 9), including controls.

The spectral mean fluorescence of maize kernel components (germ and endosperm) incubated in *A. flavus* inoculum at three time points over a 9 day period (Days 5, 7, and 9) is presented in **Figures 5A–C**. In a similar manner, as mentioned earlier, imaging data were statistically analyzed to determine the existence of group differences in spectral peak shifts between the two fungal inoculi and the controls, in the germ (**Figure 5D**) and the endosperm (**Figure 5E**). There was a marginally significant (*p* = 0.056) main effect of treatment evident in the germ on day 5 (**Figure 5D**). The Tukey-Kramer *post hoc* pairwise test determined a significant difference between the AF13 treated and the control groups. There was no significant difference between spectral peaks of the AF36 and the other two treatment groups. Day 7 showed no significant main effect or group differences in the germ. On day 9 there was a significant main effect, *p* < 0.001, of treatment, with a significant difference between the peaks of the AF13 and the AF36 treatment groups as well as between the controls and the AF36 treatment group (**Figure 5D**). There was no significant peak difference between the AF13 treated and the control groups.

A significant (*p* = 0.029) main effect of treatment existed in the endosperm on day 5 (**Figure 5E**). The *post hoc* analysis determined a significant peak difference between the AF36 treated and the control groups. There was no significant difference between the two treatment groups, or between the AF13 treated and the controls. Similarly, as seen in the germ, there were no significant peak differences between any of the groups on day 7. The statistical analysis of data in the endosperm on day 9 mimicked the findings of the germ. There was a main effect of treatment (*p* < 0.001), with the main difference exhibited by the peaks of the AF36 group compared to both the AF13 and the control groups (**Figure 5E**). As before, there was no evident difference between spectral peaks in the control group and the AF13 group.

Overall, the analysis of the results from the maize kernel components indicated that on day 5 AF13 was detected in the germ and AF36 in the endosperm, based on the position of the fluorescence peak shifts which were significantly different from the controls at that time point. Although not conclusive, these observations may potentially indicate a separation of strain colonization where the toxin producing strain colonized the germ first, presumably because aflatoxin production requires nutrients more readily available in the germ, before proceeding to colonize the rest of the kernel including the endosperm where it was detected on day 9. It appears that the atoxigenic AF36 may have proceeded directly to the endosperm where it was detected on day 5, before proceeding to colonize the rest of the kernel including the germ where it was detected on day 9 with a significant fluorescence peak shift from both the controls and the toxigenic AF13. Although on day 9 the AF13 and AF36 peak shifts were significantly different from each other, it was not possible to differentiate the control and AF13 fluorescence peaks. However, the intensity of the AF13 fluorescence in the endosperm was approximately double that of AF36. This observation may provide an additional component for the detection strategy when attempting to separate the toxigenic from the atoxigenic *A. flavus* fluorescence signals with spectral fluorescence.

### Chemical Data – Germ and Endosperm

**FIGURE 6:**
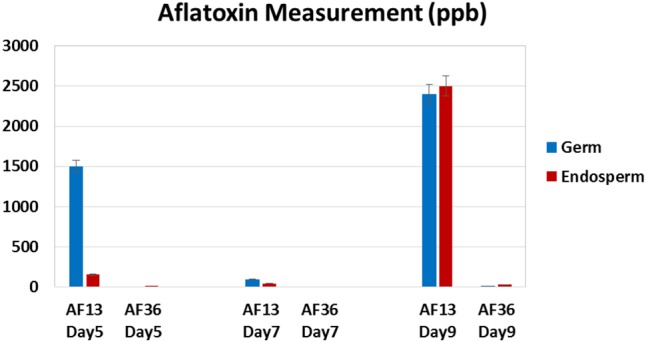
Aflatoxin content (parts per billion, ppb) of maize kernel components (germ and endosperm) inoculated with toxigenic (AF 13) and atoxigenic (AF 36) *A. flavus* at three time points over a 9 day period (Days 5, 7, and 9). Error bars represent ± 5%.

Aflatoxin concentrations of the maize kernel components (germ and endosperm) in ppb (μg/kg) are presented in **Figure 6**. As seen previously in literature (Fennell et al., 1973; Diener et al., 1987; Rajasekaran et al., 2008; Hruska et al., 2014), aflatoxin production starts in the germ and progresses rapidly throughout the kernel. On day 5 most of the aflatoxin was detected in the germ with very little detected in the endosperm. On day 7 the endosperm contained approximately half the amount of aflatoxin as did the germ. Finally, on day 9 the germ and the endosperm contained similar concentrations of aflatoxin.

As expected of an atoxigenic strain, AF36 showed no aflatoxin production on day 5 or 7. A minimal amount of aflatoxin was detected on day 9, presumably due to environmental cross-contamination.

## Discussion

The present study examined the internal fluorescence spectral emissions from cross-sections of kernels infected with both toxigenic and atoxigenic *A. flavus* in order to gain a deeper understanding of the interaction between the fluorescence signal and fungal invasion resulting in the production of aflatoxin. In addition, the internal components of the kernels (germ and the endosperm) were examined (imaged and chemically analyzed) to determine the major areas of aflatoxin accumulation and progression over time.

Although there was no discernible difference between the aflatoxin producing *A. flavus* from the non-producing strain in maize cross-sections on the basis of a fluorescence peak shift, the marked difference in fluorescence intensity found on day 9 post-inoculation, between the two strains, may be a useful indicator of the location of aflatoxin contamination. Results also reveal a possible preferential difference in the internal colonization strategy of maize kernels between the toxigenic and atoxigenic strains of *A. flavus*, suggesting a potential temporal window for the possibility of differentiating the strains based on fluorescence spectra.

Analysis of the kernel component data indicates that the toxin producing AF13 was detected in the germ and the atoxigenic AF36 in the endosperm on the fifth growth day based on the fluorescence peaks shifts which were significantly different from the controls at that time point. This may indicate a separation of strain colonization where the toxin producing strain colonized the germ first, presumably because aflatoxin production requires nutrients more readily available in the germ, before proceeding to colonize the rest of the kernel including the endosperm where it was detected on the ninth day of growth. The observation that aflatoxin producing fungi preferentially colonize the germ tissue of maize kernels has been reported by others (Keller et al., 1994; Mellon et al., 2005). A study that utilized colored mutants of aflatoxin producing fungi preferentially invaded the germ and the aleuronic layer of non-germinating kernels (Keller et al., 1994). This is in agreement with the substrate utilization study in whole kernels where it was found that *A. flavus* selectively utilized sucrose and raffinose, free sugar reserves, before utilizing lipid reserves with the premise that most free sugars and fats are located in the germ (Mellon et al., 2005). Because free saccharides seem to be prerequisite for the initiation of aflatoxin biosynthesis, the fungus invades the germ for an easy access to carbon substrates (Mellon et al., 2005).

Liu et al. (2016) also reported an association between aflatoxin biosynthesis and certain nutrients present in the germ and endosperm of maize kernels. The study revealed that sucrose, glucose, maltose, arginine, glutamate, aspartate, and zinc induced biosynthesis of aflatoxin B_1_ in *A. flavus* NRRL 3357 and may be the key nutrients in aflatoxin production overall as all of these nutrients were abundant in defatted substrates including maize germ. Not surprisingly, the amino acid concentration in the maize germ was 2- to 5-fold higher than in the endosperm indicating a differential role of the nutrients in the two substrates. The highest aflatoxin was induced by glutamate and aspartate, followed by arginine. In defatted maize germ, arginine was the dominant amino acid (Liu et al., 2016). The effects of amino acids on aflatoxin induction have been studied previously. It has been reported that proline significantly stimulated production of aflatoxin by *A. flavus* (Payne and Hagler, 1983; Wilkinson et al., 2007). Since glutamate is the source of proline and arginine (Winter et al., 2015), and both glutamate and proline are nitrogen sources for stimulating aflatoxin production (Yu, 2012), it is not unreasonable to infer a similar effect of arginine on aflatoxin biosynthesis in maize germ. Several reports noted the importance of trace elements, particularly zinc, in stimulating aflatoxin in liquid media (Mateles and Adye, 1965; Reddy et al., 1971; Marsh et al., 1975). An abundance of zinc was also found in defatted maize germ (Lillehoj et al., 1974; Liu et al., 2016). Studies that added trace elements to *A. flavus* infected defatted maize germ found significant increase in aflatoxin due to several minerals including zinc, manganese, and copper (Lillehoj et al., 1974), also found naturally in uninfected defatted maize germ (Lillehoj et al., 1974; Liu et al., 2016). The presented evidence largely supports the idea of the preferential colonization of maize germ by aflatoxigenic *A. flavus* due to the presence of readily available nutrients essential for the production of aflatoxins, suggested by the current study (**Figures 5, 6**).

Over 80% of the maize endosperm is composed of starch (Liu et al., 2016). Starch has not been shown to stimulate aflatoxin production (Liu et al., 2016), whereas fungal growth has been shown to be mainly propagated by sugars and starches. In the present study, the atoxigenic AF36 may have initially avoided the germ and proceeded directly to the endosperm where it was detected on day 5, before colonizing the rest of the kernel including the germ, where it was detected on day 9 with a significant fluorescence peak shift from both the controls and the toxigenic AF13.

It appears that toxigenic and atoxigenic *A. flavus* differ in how each secures resources for survival. The toxigenic strain may rely on its secondary metabolic pathway to synthesize aflatoxin and utilizes the ample and readily available stores of energy, nitrogen, and carbon sources provided by simple sugars, amino acids, and minerals in the maize germ which are essential for production of aflatoxin (Liu et al., 2016). After the stores are depleted due to initial mycelial and conidial growth and aflatoxin synthesis in the germ, the fungus presumably proceeds to utilize the starch reserves as it colonizes and infects the rest of the kernel. The progress of a toxigenic strain of *A. flavus* through maize kernels was demonstrated by utilizing green fluorescence protein (GFP) labeled toxin producing strain AF70 to monitor progress of the fungus in kernels over 9 days of growth (Hruska et al., 2014). The fungus was present in the germ near the pedicel and black layer on the third day post-inoculation and continued to spread in the germ through day 5. By the ninth day post-inoculation, the fungus moved beyond the kernel germ and encroached on the entire endosperm demonstrated by intense internal fluorescence (Hruska et al., 2014), a phenomenon replicated in the current study (**Figures 4C, 5C**).

Since growth propagation seems to be fueled by starch (Liu et al., 2016), the atoxigenic fungi may not have a preference for the germ tissue, but instead may gain access to the kernel endosperm from a random point of entry and rapidly colonize the kernel outside as well as inside while successfully compromising the integrity of the invaded kernel (Hruska et al., 2014).

Regardless of the route of invasion, eventually both types of fungi spread over the whole maize kernel demonstrated by natural fluoresce present in the endosperm. However, the nature of fluorescence in the germ may differ between the toxigenic and atoxigenic fungal strains. Lillehoj et al. (1976) noticed that some field maize kernels contaminated with aflatoxin exhibited the characteristic BGY fluorescence in a narrow area around the germ and a glow directly under the seed coat (the aleurone layer). Once the seed coat was removed, an intense, slightly more yellow fluorescence than the typical BGYF was revealed in the endosperm. When some of these intensely fluorescing kernels were analyzed chemically, some contained detectable amounts of aflatoxin, but others did not (Lillehoj et al., 1976). This is a phenomenon also noted in our laboratory and may reflect the presence of non-toxin producing, but fluorescing fungal strains and may account for the incidence of false negative results of an aflatoxin test. A false negative test is much more difficult to remedy. In the present experiments aflatoxin was detected on the fifth growth day in the germ of the separated kernel components (**Figures 5D, 6**) and cross-sections (**Figure 4D**). However, the fluorescence is not always apparent through the pericarp and the length of infection is difficult to pinpoint in the field or in storage.

In the present study, by day 9 of incubation, the AF13 and AF36 peak shifts inside the kernel components were significantly different from each other, but it was not possible to differentiate the control and AF13 fluorescence peaks. However, the intensity of the AF13 fluorescence in the endosperm was approximately double and in the germ it was approximately half that of AF36, which remained in the vicinity of the controls (**Figures 5C–E**). The differences in intensity in all groups on days 5 and 7 were insignificant compared to the drastic changes of AF 13 on day 9. Seemingly, by day 9 the atoxigenic fungus could be separated from the control and the toxigenic fungus in both the endosperm and in the germ, on the basis of a peak wavelength shift, and the toxigenic fungus could be separated from the control based on the fluorescence intensity. Therefore, both peak shift and intensity as well as timing may be essential in distinguishing toxigenic and atoxigenic fungi based on spectral features. These observations provide additional components to the detection strategy when attempting to separate the toxigenic from the atoxigenic *A. flavus* fluorescence signals with spectral fluorescence.

## Conclusion

In conclusion, the present *in situ* study noted a clear difference in fluorescence intensity on day 9 post-inoculation, between aflatoxin producing *A. flavus* and the non-producing strain in maize cross-sections and also in the kernel components. There was also a significant shift in fluorescence peak wavelengths observed 9 days post-inoculation, allowing for the separation of AF13 and AF 36 in both germ and the endosperm. However, based on the results, it may be difficult to separate AF13 from the control unless the aforementioned intensity fluctuations are taken into consideration. The study also revealed a possible preferential difference in the internal colonization of maize kernels between the toxigenic and atoxigenic strains of *A. flavus* suggesting a potential temporal window for the possibility of differentiating the strains based on fluorescence spectra and providing data supporting the theory for the false positive phenomenon. Following studies may need to focus on further exploring the difference between the fluorescence present in the germ and the endosperm in aflatoxin contaminated and uncontaminated maize.

## Author Contributions

All authors participated in planning the experiments and offering advice on the manuscript. ZH designed the experiments, analyzed and interpreted the data, and wrote the manuscript. HY and RB oversaw the implementation of the experiments and offered advice and constructive critique of the manuscript. RK collected and processed image data and edited the manuscript.

## Conflict of Interest Statement

The authors declare that the research was conducted in the absence of any commercial or financial relationships that could be construed as a potential conflict of interest.
